# Hashimoto's Thyroiditis and Kikuchi's Disease: Presentation of a Case and Review of the Literature

**DOI:** 10.1155/2012/267595

**Published:** 2012-11-28

**Authors:** Athanasios Saratziotis, Konstantinos Karakousis, Kelly Tzika, Katerina G. Oikonomou, Panagiotis J. Vlachostergios

**Affiliations:** ^1^Department of Otorhinolaryngology, General Hospital of Larissa, 41221 Larissa, Greece; ^2^Department of Internal Medicine, General Hospital of Larissa, 41221 Larissa, Greece; ^3^Department of Biology, University of Crete, 71409 Heraklion, Crete, Greece

## Abstract

We report the case of a 19-year-old woman with a history of Hashimoto's thyroiditis who presented with tender right anterior cervical lymphadenopathy and fever. Workup for infectious, autoimmune, and malignant causes was unremarkable. Surgical removal of cervical lymph nodes after detailed magnetic resonance (MR) imaging disclosed necrotizing lymphadenitis, also known as Kikuchi's disease (KD). The patient was treated with a short-term course of steroids, due to the onset of pancytopenia and borderline antiphospholipid antibodies combined with increased anti-thyroglobulin (anti-TG) titers. Despite being a diagnosis of exclusion, KD should be included in the differential of such patients, particularly in cases of previous or concurrent autoimmune diseases such as Hashimoto's thyroiditis, which necessitate a long-term follow-up.

## 1. Introduction

Hashimoto's thyroiditis is an autoimmune disease characterized by hypothyroidism and asymmetric thyroid growth. Positive serologic testing of antithyroid peroxidase (anti-TPO) antibody and/or anti-thyroglobulin (anti-TG) antibody supports the clinical diagnosis. The disease results in single or multiple nodules or pseudonodules in the thyroid tissue.

 The presence of enlarged lymph nodes in a patient may be due to a number of clinical conditions that are grossly divided to acute infectious states, as well as lymphoproliferative and autoimmune conditions, which follow a more protracted course. In the context of an autoimmune disease such as Hashimoto's thyroiditis, clinicians should consider the possibility that lymphadenopathy, when present, may be associated to other autoimmune conditions.

## 2. Case Report

A 19-year-old woman was referred because of a 20-day history of sore throat and presence of a right anterior cervical mass, causing mild pain and tenderness over the right sternocleidomastoid muscle. She also reported intermittent fever up to 38.5°C and chills for the last 10 days before admission. The patient denied any eating difficulty, loss of weight, respiratory compromise, skin rash, or any ear pain or discharge. She was a nonsmoker, nondrinker, with a medical history of Hashimoto's thyroiditis and her current medication included levothyroxine and amoxicillin. 

 On physical examination, the patient was afebrile, with tender right cervical adenopathy and enlarged tonsils without exudate. Nasal and ear examination results were unremarkable and there was no peripheral lymphadenopathy or hepatosplenomegaly detectable. Her white blood cell count was 3.5 × 10^9^/L and consisted of 41.6% neutrophils, 10.2% monocytes, 46.2% lymphocytes, 1.7% eosinophils, and 0.3% basophils. Her hemoglobin level was 11.7 g/dL and her platelet count was 168 × 10^9^/L. A chest radiograph was negative for infiltrates or lymphadenopathy.

Several laboratory tests were performed to investigate systemic infectious and autoimmune causes of cervical lymphadenopathy. These included a peripheral smear, clotting tests, serology for infectious agents such as EBV, CMV, HIV, toxoplasmosis, erythrocyte sedimentation rate (ESR), c-reactive protein level, liver function tests, direct Coombs test, C3 and C4 levels, serum protein electrophoresis, and an autoimmune screen, including antinuclear antibodies (ANA), anti-dsDNA antibodies, anti-SSA/Ro and anti-SSB/La antibodies, and antiphospholipid antibodies. Further, a mantoux test was performed to identify any exposure to tuberculosis. Abnormal investigations were as follows: antinuclear factor titre-160 (with a fine, speckled pattern), anti-ds DNA, anti-SSA/Ro, anti-SSB/La, anti-Sm, and anti-RNP antibodies-absent, anticardiolipin IgG antibody-borderline positive. Gamma globulin level was increased and viral serology testing disclosed prior EBV and CMV infection. Thyroid function tests were normal; however, anti-TG titre was further increased compared to the baseline level before the onset of fever.

**Figure 1:**
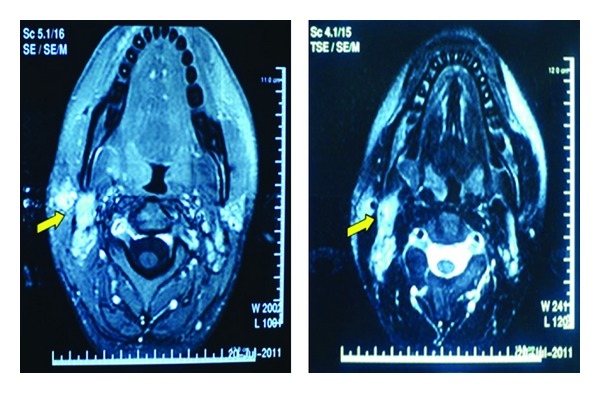
T1- and T2-weighted MR images showing right-sided cervical lymphadenopathy with focal hypointense areas with peripheral distribution and clear margins.

An ultrasound was performed to assess the solid or/and cystic component of the mass. An imaging workup of the chest and abdomen with CT was negative for mediastinal or abdominal lymphadenopathy. A magnetic resonance imaging (MRI) scan of the neck was also performed to better evaluate the mass and any accompanying soft tissue lesions and disclosed bilateral tonsillar enlargement and right cervical adenopathy extending from the angle of the jaw down to the C6 level (Figure 1).

**Figure 2:**
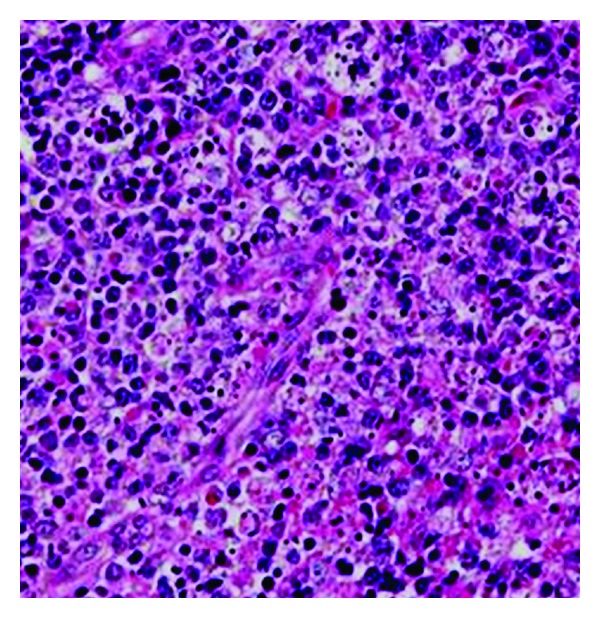
Hematoxylin and eosin stain showing central apoptotic cellular debris surrounded by histiocytes, crescentic immunoblasts, and plasmacytoid monocytes (×200).

Due to the existence of multiple cervical nodes with not so well-defined margins, a selective neck dissection with bilateral tonsillectomy was performed. The pathology report disclosed histiocytic necrotizing lymphadenitis. Within the lymph node cortex there were areas showing central apoptotic cellular debris surrounded by histiocytes, crescentic immunoblasts, and plasmacytoid monocytes (Figure 2). 

Neutrophils were absent and eosinophils and plasma cells were inconspicuous. Histiocytes stained positively with antibodies to CD68 and myeloperoxidase. Within necrotic areas there were predominantly CD3+ and CD8+ T lymphocytes. Immunoblasts were also of T-cell origin, with positive CD3 and CD43 staining but no expression of TdT or CD34. Examination of tonsillar tissue showed reactive hyperplasia without evidence of lymphoma or carcinoma. Histopathological results were compatible with a diagnosis of Kikuchi-Fujimoto disease.

The patient continued to have less intense fever for 3 days after admission, not exceeding 38°C. In addition, in the first postoperative day, the patient developed pancytopenia. Her white blood cell count was 2.8 × 10^9^/L and consisted of 50.3% neutrophils, 4.5% monocytes, 43.3% lymphocytes, 1.6% eosinophils, and 0.3% basophils. Her hemoglobin level was 11.1 g/dL and her platelet count was 137 × 10^9^/L. A direct Coombs test was negative and bone marrow biopsy revealed decreased bone marrow cellularity without infiltration or fibrosis. Given the patient's clinic-hematological profile and borderline positivity for antiphospholipid antibodies (APLAs), a short 2-month course of methylprednisolone was prescribed, at a dose of 32 mg daily, after which she recovered fully and her blood count returned to normal. The patient remains asymptomatic and APLA-negative, ten months after initial diagnosis, and continues to be regularly followed-up.

## 3. Discussion

Kikuchi's disease (KD), also known as histiocytic necrotizing lymphadenitis or Kikuchi-Fujimoto disease, is a rare clinical entity affecting predominantly young women of Asian descent and involves noncancerous enlargement of the lymph nodes. Most patients present with unilateral tender cervical lymphadenitis, although any lymph node region may be involved. Fever often accompanies adenopathy, whereas hepatosplenomegaly and constitutive symptoms including headache, weight loss, fatigue, chills, night sweats, and gastrointestinal complaints rarely occur. Various types of skin rashes (maculopapular, urticarial) have also been reported [7].

No specific laboratory findings have been described for KD. Leukopenia may be present in a significant proportion of patients. The presence of atypical lymphocytes and/or positive serology for EBV in some reports suggests that the latter might be implicated in the aetiopathogenesis of the disease. KD has also been associated with the presence of autoantibodies indicative of SLE or other autoimmune diseases, including Hashimoto's thyroiditis, as in our case [1–6]. All previously reported cases of KD associated with Hashimoto's thyroiditis are presented in Table 1.

In contrast to the majority of the above described cases, our patient had a previous history of Hashimoto's thyroiditis, and the levels of anti-TG antibodies were found to be gradually increasing during several measurements, weeks before the onset of symptoms. In a small analysis of 22 cases of cervical lymphadenopathy associated with Hashimoto's thyroiditis with the use of fine needle aspiration cytology (FNAC), none of the lymph node aspirates was in favor of KD [8].

 Radiographic findings from ultrasonography and CT, although not specific for KD, are often helpful for obtaining tissue diagnosis. MRI being a more sensitive tool for detection of increased cellularity, extracellular free water, hemorrhage, fibrosis, fat, and metal deposition might be useful for early diagnosis of KD. Enlarged lymph nodes usually do not exceed 2.5 cm, and focal nonenhancing areas suggestive of necrosis are occasionally found within nodes. T2-weighted images may reveal focal hypointense areas, frequently with a peripheral distribution and clear margins, which presumably represent histopathological findings of coagulative necrosis in paracortical areas. Accordingly, KD should be considered when T2-weighted images demonstrate hypointensity areas at the peripheries of enlarged cervical nodes [9].

 The definitive diagnosis of KD is made through lymph node excision biopsy and histologic examination. There are several classic histologic features of KD. The affected lymph nodes have patchy necrotizing regions, particularly in the paracortical areas. They often contain well-circumscribed areas of eosinophilic fibrinoid material with a substantial degree of karyorrhexis. Transformed lymphocytes (immunoblasts) may surround the necrotic areas, creating a characteristic mottled appearance at low magnification. Nuclear debris (nuclear dust) is evenly scattered throughout the necrotic areas and is associated with atypical mononuclear cells, which may be macrophages phagocytosing the debris. Another consistent histologic observation is the absence of granulocytes and plasma cells [7].

KD usually follows a benign course, with spontaneous resolution of fever and lymphadenopathy in the majority of patients. However, a concurrent autoimmune disease or the risk of developing an autoimmune disease requires repeated autoantibody testing and long-term follow-up of the subgroup of patients, especially women, with positive autoantibodies at initial presentation [10]. Patients with recurrent episodes are more likely to have fever and fatigue with extranodal involvement and remain symptomatic for a rather longer duration. The significantly higher positive rate of the fluorescence antinuclear antibody (FANA) test in such patients compared to the nonrecurrent group might support its use as a predictor of recurrence, if prospectively confirmed [11].

## Figures and Tables

**Table 1:** Summary of cases of Kikuchi's disease (KD) associated with Hashimoto's thyroiditis (HT).

Reference	Age	Gender	Onset of KD related to HT	Clinical presentation	Autoantibody screen	Treatment	Evolution
[1]	17	Female	Concurrent KD, HT	Fever, cervical lymphadenopathy, goiter, hepatosplenomegaly, and urticaria	Anti-TPO, ANA, anti-dsDNA, anticardiolipin, low C3, C4	Propranolol, prednisone, and hydroxychloroquine	Improvement after 3 months, asymptomatic 1 year after diagnosis, continues treatment

[2]	N/A	N/A	Concurrent KD, HT	Cervical lymphadenopathy, goiter	N/A	N/A	N/A

[3]	N/A	N/A	KD and HT	N/A	N/A	N/A	N/A

[4]	26	Female	Concurrent KD, HT	Odynophagia, fever, malaise, and cervical lymphadenopathy	Negative	Supportive	Complete resolution after 12 days

[5]	26	Female	Concurrent KD, HT	Fever, cough, weight loss, emesis, abdominal pain, cervical, mediastinal and abdominal lymphadenopathy, and hepatosplenomegaly	ANA, low C3, C4	Prednisone	Improvement after 1 week, asymptomatic after 6 months

[6]	30	Female	KD in patient with history of HT	Cervical lymphadenopathy, trismus, and fever	Not performed	Ibuprofen	Complete resolution after several days
